# A COLLABORATIVE AUTOETHNOGRAPHY OF FOREIGN-BORN FACULTY IN THE FIELD OF AGING

**DOI:** 10.1093/geroni/igac059.2869

**Published:** 2022-12-20

**Authors:** Chih-ling Liou, Karel Kalaw

**Affiliations:** Kent State University, North Canton, Ohio, United States; University of Central Oklahoma, Edmond, Oklahoma, United States

## Abstract

We draw from our personal insights as foreign-born females in the U.S. to explore how our minority status has affected our experiences/identities as faculty in the field of aging. We practiced intersectionality by considering how our understandings of “foreignness” in academia and the aging field are intertwined with other markers of difference, including race, national origin, language, and academic programs. We also draw from tenets of collaborative autoethnography to engage two autoethnographies from two countries in Asia to pool their lived experiences and collaboratively analyze and interpret them for commonalities and differences. We begin by sharing stories about graduate study pursuits and becoming faculty in the U.S. We note how our foreignness shapes our lives as aging scholars in the U.S. academy and our personal views on aging. After exchanging our first set of writings, we identified key experiences to focus on in the subsequent writing periods. Our minority backgrounds, teaching aging subjects, and using qualitative methodology are shared identification. However, our personal stories within the U.S. society and within the academy also diverge our identities and aging experiences from each other. Moreover, the themes that deal directly with identity development and the perception of aging provide a deep understanding of aging among the non-native-born population. This study highlights the value of collaborative autoethnography as a method of inquiry and reflection. Findings demonstrate that non-native-born female faculty in the field of aging faced multi-faceted challenges in both professional and personal realms. Implications for supporting foreign-born female aging scholars are discussed.

